# Impaired Learning From Negative Feedback in Stimulant Use Disorder: Dopaminergic Modulation

**DOI:** 10.1093/ijnp/pyab041

**Published:** 2021-07-01

**Authors:** Tsen Vei Lim, Rudolf N Cardinal, Edward T Bullmore, Trevor W Robbins, Karen D Ersche

**Affiliations:** 1 Department of Psychiatry, University of Cambridge, Cambridge, UK; 2 Institute of Systems Neuroscience, University Medical Center Hamburg-Eppendorf, Hamburg, Germany; 3 Cambridgeshire and Peterborough NHS Foundation Trust, Cambridge, UK; 4 Department of Psychology, University of Cambridge, Cambridge, UK

**Keywords:** Addiction, cocaine, hierarchical Bayesian modeling, punishment, reinforcement learning, reward

## Abstract

**Background:**

Drug-induced alterations to the dopamine system in stimulant use disorder (SUD) are hypothesized to impair reinforcement learning (RL). Computational modeling enables the investigation of the latent processes of RL in SUD patients, which could elucidate the nature of their impairments.

**Methods:**

We investigated RL in 44 SUD patients and 41 healthy control participants using a probabilistic RL task that assesses learning from reward and punishment separately. In an independent sample, we determined the modulatory role of dopamine in RL following a single dose of the dopamine D_2/3_ receptor antagonist amisulpride (400 mg) and the agonist pramipexole (0.5 mg) in a randomised, double-blind, placebo-controlled, crossover design. We analyzed task performance using computational modelling and hypothesized that RL impairments in SUD patients would be differentially modulated by a dopamine D_2/3_ receptor antagonist and agonist.

**Results:**

Computational analyses in both samples revealed significantly reduced learning rates from punishment in SUD patients compared with healthy controls, whilst their reward learning rates were not measurably impaired. In addition, the dopaminergic receptor agents modulated RL parameters differentially in both groups. Both amisulpride and pramipexole impaired RL parameters in healthy participants, but ameliorated learning from punishment in SUD patients.

**Conclusion:**

Our findings suggest that RL impairments seen in SUD patients are associated with altered dopamine function.

**Significance Statement**

One of the defining aspects of drug addiction is the continued use of drugs despite their harmful effects that perpetuate over time. Chronic drug use alters the dopamine system, and this is widely believed to account for such maladaptive behaviour by impairing the ability to learn from consequences. Here, we investigated reinforcement learning in people addicted to stimulant drugs with a task that separately measures learning from reward and punishing feedback. We then analyzed these data using computational modelling, a method that is sensitive to subtle changes in learning. Our analysis showed that stimulant-addicted patients have a selective impairment in learning from negative feedback. In a subsequent study, we showed that the pharmacological modulation of dopamine D_2/3_ receptors improved stimulant users’ difficulties learning from negative feedback, thus supporting the dopamine dysfunction hypothesis. Our findings provide novel insights into why stimulant-addicted patients often fail to learn from their negative experiences.

## Introduction

Stimulant drug addiction, or stimulant use disorder (SUD), is a major public health problem that causes significant harm to individuals, their families, and society (Degenhardt et al., 2014). The behavior of chronic stimulant drug users often seems maladaptive and ill-judged, as they frequently behave in ways that are detrimental to their own interests, regardless of the negative consequences. One possibility is that drug-induced neuroadaptations may change how individuals learn from the consequences of their actions, an impairment that might extend beyond drug-taking (Maia and Frank, 2011).

Reinforcement learning (RL) is an influential account of adaptive instrumental behavior that provides a normative framework of how humans use past consequences to guide future behavior (Sutton and Barto, 1998). Optimal RL includes multiple processes such as valuation, reward prediction, and action selection (Niv, 2009), and many of these processes are suggested to be modulated by dopamine (Bayer and Glimcher, 2005; Frank et al., 2007; Steinberg et al., 2013), a neurotransmitter affected by stimulant drugs such as cocaine and amphetamine. Chronic stimulant drug use has been associated with a downregulation in dopamine neurotransmission in fronto-striatal circuits (Volkow et al., 2004) that underpin learning and value-based decision-making (Ernst and Paulus, 2005; Glimcher, 2011; O’Doherty et al., 2017). Animal studies shown that cocaine exposure disrupts key aspects of RL, including reward prediction (Burton et al., 2018; Takahashi et al., 2019) and reinforcement value (Schoenbaum and Setlow, 2005; Groman et al., 2020). Although similar observations have also been reported in human stimulant drug users (Harlé et al., 2015; Parvaz et al., 2015; Ersche et al., 2016), the exact profile of impairments remains elusive. While it is widely assumed that impairments in RL in SUD patients are dopaminergic in nature, it is unclear how these disruptions are modulated by dopaminergic agents. There is some evidence for modulatory effects of dopamine manipulations on cognitive dysfunction in SUD (Ersche et al., 2010, 2011a; Goldstein et al., 2010). However, compared with control participants, SUD patients show different behavioral and neural responses following dopaminergic drug challenges, suggesting that such medication alters RL differentially in SUD patients (Volkow et al., 2005; Ersche et al., 2010, 2011a; Goldstein et al., 2010). The precise actions of dopaminergic drugs are difficult to determine in human studies, but drug challenges may provide insight into the neurochemical underpinnings associated with RL in SUD patients.

A conventional approach to quantify RL performance is to compute summary scores that reflect performance accuracy and analyze them with a frequentist approach (e.g., Strickland et al., 2016). As RL impairments can also result from latent processes that are not directly measured by summary scores, such as motivational deficits, slower contingency learning, or inconsistencies in choice behavior, complementary approaches are needed. An increasingly popular method is to use computational models to describe RL, allowing the quantification of latent RL parameters (Sutton and Barto, 1998). Individual differences in RL are then reflected in model parameters, which can be compared between groups (Daw, 2011). Although simple RL models might not perfectly capture all the RL-related cognitive processes, the model parameters can provide sensitive behavioral measures (Heinz et al., 2016; Robbins and Cardinal, 2019).

Here, we combine both conventional and computational approaches to address the following objectives: (1) to characterize the RL profile in a large community sample of SUD patients using a behavioral task that assesses learning from reward and punishment separately; (2) to explore the modulatory effects of a dopamine D_2/3_ receptor agonist and an antagonist on RL in an independent sample of SUD patients. We used 2 pharmacological agents that selectively target the D_2/3_ system: the dopamine receptor antagonist amisulpride and the dopamine receptor agonist pramipexole (Wright et al., 1997; Rosenzweig et al., 2002). For the computational analysis, we employed a well-established RL model (Watkins and Dayan, 1992) and adopted the learning rate, the impact of reinforcement on choices, as our key outcome measure. We also modeled other processes that support learning, such as the extent to which behavior is motivated by learned values (reinforcement sensitivity) and tendency to perseverate. We hypothesized that these latent learning parameters are impaired in SUD patients and would be modulated differentially by dopamine agonist and antagonist agents. Since SUD patients have abnormal dopamine transmission, we predicted that these dopaminergic agents would modulate RL performance differentially in SUD patients compared with healthy controls.

## Methods

We studied two independent samples of stimulant-addicted individuals and matched healthy volunteers. For inclusion, participants had to be at least 18 years old and able to read and write in English. Stimulant drug users needed to meet the DSM-IV-TR criteria for stimulant drug dependence (American Psychiatric Association, 2000), whereas control participants had to be healthy without a personal history of substance use disorders. Participants were recruited from the local community in Cambridge (UK) by advertisement and word of mouth. Both studies were approved by a Cambridge Research Ethics Committee. All participants provided written informed consent prior to enrollment and were screened for psychiatric disorders using the Mini-International Neuropsychiatric Inventory (Sheehan et al., 1998); psychopathology in drug users was further evaluated using the Structured Clinical Interview for DSM-IV (First et al., 2002). All SUD patients were actively using stimulant drugs, which was confirmed by positive urine screens prior to testing, suggesting that they had been using the drug within the past 72 hours. All urine samples provided by control participants tested negative for all drugs; participants were also breathalyzed to verify sobriety. Exclusion criteria for all participants included a lifetime history of a psychotic disorder, neurological illness or traumatic head injury, and acute alcohol intoxication. All participants completed the National Adult Reading Test (Nelson, 1982) and the Barratt Impulsiveness Scale (Patton et al., 1995) to estimate the verbal intelligence quotient (IQ) and impulsive personality traits, respectively. Participants also reported their monthly disposable income and rated their willingness to pick up £0.50 off the floor on a visual analog scale (always—never) as a proxy for the subjective value of monetary reward. SUD participants additionally completed the Obsessive-Compulsive Drug Use Scale (Franken et al., 2002) as a measure of compulsive drug use.

### Study 1

#### Sample

Forty-four men who met the DSM-IV-R criteria for cocaine dependence, referred to as cocaine use disorder (CUD), had been using cocaine for a mean of 13.7 years (SD = ±8.0) and the majority also met the criteria for dependence on another substance (55% opiates, 7% alcohol, 14% cannabis). Participants with co-morbid opiate dependence were either prescribed methadone (32%; mean daily dose = 49 mg, SD = ±13.0) or buprenorphine (18%; mean daily dose = 7.5 mg, SD = ±3.5). Some CUD patients were taking prescribed medication, including antidepressants (14%), benzodiazepines (9%), painkillers (16%), antibiotics (5%), and anticoagulants (5%). The 41 healthy control participants did not use prescribed mediation and reported low levels of drug and alcohol use, as reflected in low total scores on the Alcohol Use Disorder Identification Test (Saunders et al., 1993) (mean score = 3.4, SD = ±1.7) and Drug Abuse Screening Test (Skinner, 1982) (mean score = 0.08, SD = ±0.3). CUD patients reported a significantly lower monthly disposable income than controls (t_83_ = 2.6, *P* = .012; see Table 1).

**Table 1.** Sample Demographics and Task Performance of the Two Studies.

	Study 1		Study 2	
Groups	Control	CUD	Control	SUD
Demographics	Mean (SD)	Mean (SD)	Mean (SD)	Mean (SD)
Task performance	Mean (SD)	Mean (SD)	Mean (SD)	Mean (SD)
Sample size (n)	41	44	18	18
Age (years)	40.1 (12.6)	40.9 (9.2)	32.7 (6.9)	34.3 (7.2)
Gender (% male)	100	100	83	83
Verbal IQ (NART score)	115 (6.2)	103 (7.1)	108 (6.0)	109 (8.1)
Disposable income (£/month)	657 (501)	387 (462)	470 (389)	621 (866)
Subjective value of 50 pence (% rating)	81.5 (23.0)	87.1 (19.6)	72.9 (31.0)	87.4 (18.8)
Trait impulsivity (BIS-11, total score)	56.1 (6.7)	79.5 (11.4)	62 (7.2)	82 (9.5)
Duration of stimulant drug use (years)	—	13.7 (8.0)	—	12.3 (6.7)
Compulsive drug use (OCDUS total score)	—	34.1 (10.1)	—	25.6 (7.9)
Total % correct (reward)				
Placebo	73.0 (21.6)	57.9 (22.5)	87.1 (24.5)	81.1 (18.6)
Amisulpride	—	—	87.9 (23.7)	75.8 (27.3)
Pramipexole	—	—	75.7 (30.9)	61.8 (35.2)
Total % correct (punishment)				
Placebo	63.6 (12.0)	54.3 (10.6)	73.3 (19.3)	61.7 (13.5)
Amisulpride	—	—	78.5 (17.5)	62.4 (19.8)
Pramipexole	—	—	72.9 (15.2)	64.7 (18.8)

Abbreviations: BIS-11, Barratt Impulsiveness Scale; CUD, cocaine use disorder; NART, National Adult Reading Test; OCDUS, Obsessive-Compulsive Drug Use Scale; SUD, stimulant use disorder.

#### RL Task

**Figure 1.**
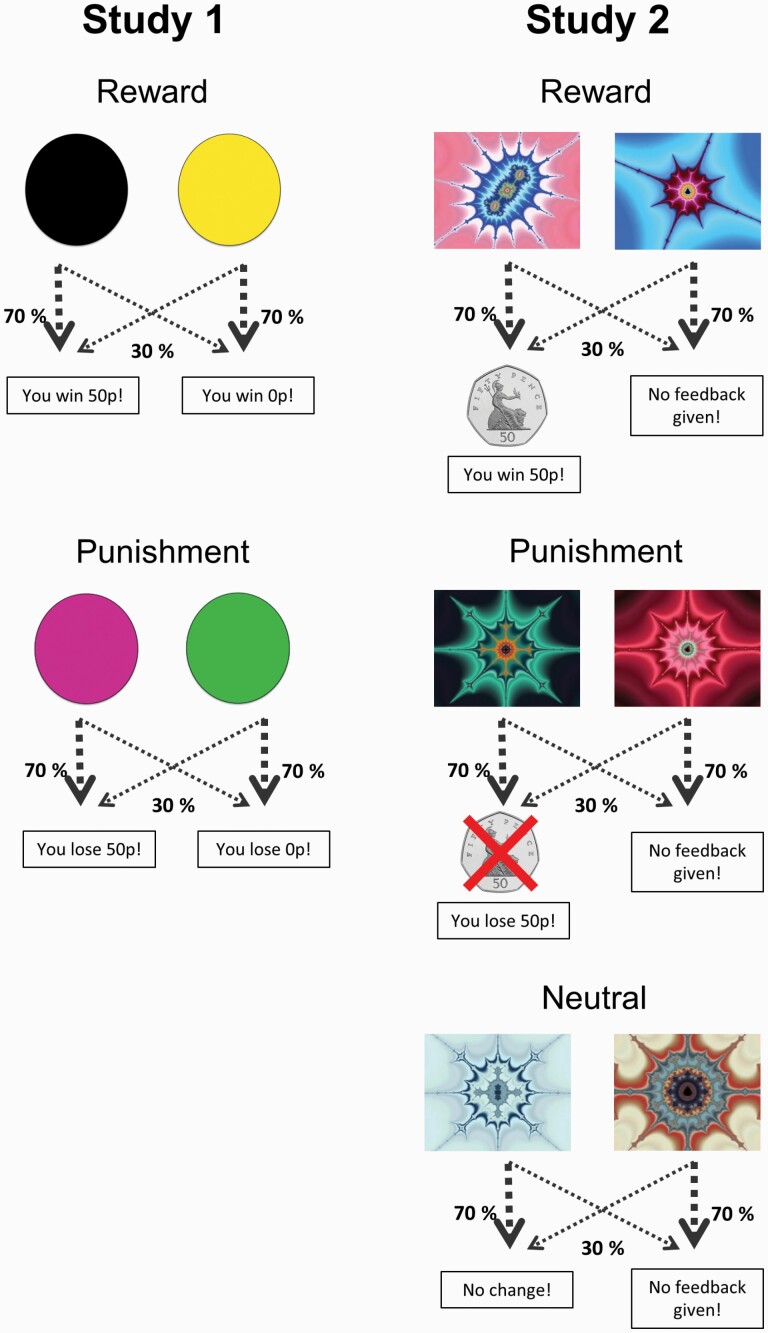
Schematics for the probabilistic reinforcement learning task of study 1 and study 2. In each trial, participants were first presented with a pair of stimuli and required to select 1 stimulus. After selection, the computer presented an outcome phrased in terms of monetary gains (positive) or losses (negative); this allowed the separate assessment of learning from reward and punishment. In both studies, each condition was differentiated by unique stimulus pairs and feedback and interspersed across 120 trials and presented in a randomized order. Optimal choices are reinforced 70% of the time, so participants needed to accrue experience over time to determine the choices that would maximize their financial gains and minimize their losses.

Our task evaluated learning from financial gains and losses (Bland et al., 2016) (Figure 1). Participants were presented with pairs of colored circles and asked to learn by trial and error to select the stimulus that maximized their overall earnings. The 2 conditions of reward and punishment were differentiated by feedback. Specifically, feedback was explicitly framed as wins (“you win 50 pence” and “you win 0 pence”) and losses (“you lose 50 pence” and “you lose 0 pence”) in the reward and punishment conditions, respectively. Participants completed 120 learning trials, with each reinforcement condition represented by unique stimulus pairs and repeated 60 times, interspersed randomly throughout the task. Optimal choices for each stimulus pair were reinforced 70% of the time either by winning £0.50 (reward) or avoid losing £0.50 (punishment).

### Study 2

#### Sample

Thirty-six volunteers were recruited from the community: 18 fulfilled the DSM-IV-TR criteria for stimulant drug dependence (10 cocaine, 8 amphetamine), referred to as SUD henceforth. The remaining 18 recruits were healthy with no personal drug-taking history. SUD patients had been using stimulant drugs for an average of 12.3 years (SD = ±6.7), had no comorbid dependencies, and were not taking prescribed medication. The two groups did not differ in their disposable income (t_33_ = −0.66, *P* = .514). Data from this sample have been published elsewhere (Ersche et al., 2010, 2011a, 2011b; Kanen et al., 2019).

#### RL Task

This task has a similar design to that of study 1 but has 3 different conditions distinguished by distinct stimulus pairs and outcomes: reward, punishment, and neutral (Murray et al., 2019). Specifically, outcomes for the reward, punishment, and neutral conditions were intentionally phrased as monetary gains (i.e., you win 50 pence), losses (i.e., you lose 50 pence), and no financial consequences (i.e., no change), respectively. Unlike study 1, reward omission (i.e., win 0 pence) and punishment avoidance (i.e., lose 0 pence) were not explicitly signaled during the feedback phase; participants did not receive any explicit feedback for these outcomes (Figure 1). There was one stimulus pair per condition, each repeated 40 times in randomized order. Optimal choices for each stimulus pair were also reinforced 70% of the time.

#### Drug Administration

Participants were administered a single dose of 400 mg amisulpride or 0.5 mg pramipexole in a double-blind, placebo-controlled, crossover design. Prior to each drug administration, participants also took a dose of domperidone (30 mg), a peripheral dopamine D_2_ receptor antagonist, as a pre-treatment to the potential side effect of nausea/vomiting. We initially administered pramipexole at a dose of 1.5 mg to the first 6 participants (3 SUD and 3 control participants), which was tolerated by SUD but not by control participants. These control participants were subsequently administered 0.5 mg pramipexole on a separate session, which was well-tolerated. Thereafter, all remaining participants received 0.5 mg pramipexole. In total, we included data from 18 control and 18 SUD participants, but we subsequently excluded the 3 SUD participants who received a higher dose of pramipexole from the analysis. Participants completed the RL task approximately 1.5 hours after dosing and blood samples were drawn at 1 and 2.5 hours post-dosing.

### Statistical Analyses

#### Conventional Analyses

Demographic and performance data were analyzed using SPSS v25 (IBM). We computed accuracy scores for the RL tasks, defined as the proportion of optimal choices made in 10-trial blocks. We used ANOVA models with a 2-tailed alpha value of .05, with trial block and condition (reward vs punishment) as within-subject factors and group (control vs SUD) as a between-subjects factor. We decided a priori to analyze the effects of amisulpride and pramipexole separately.

#### Computational Analyses

To examine latent learning parameters, we modeled trial-by-trial choice values using a delta-rule learning algorithm (Rescorla and Wagner, 1972), with the final choice selection process following a softmax rule (Sutton and Barto, 1998). Details of modeling procedures are reported in the supplementary Material. In its simplest form, a model consists of two parameters: learning rate (impact of feedback on choice values) and reinforcement sensitivity (how much choice values motivate actual behavior). Since different neural systems are thought to subserve learning from different valences (Pessiglione and Delgado, 2015), we decomposed the learning rate by the feedback received on that trial. For example, if a participant receives a reward (“you win 50 pence”) or a punishment (“you lose 50 pence”), we modeled that trial with the learning rate from reward and punishment, respectively, whereas trials with a reward omission (“you win 0 pence”) or punishment avoidance (“you lose 0 pence”) feedback were modeled with the learning rate from non-reward and non-punishment, respectively. However, it is not possible to model the learning rate from non-reward or non-punishment in study 2, because reward and punishment omission feedback were not explicitly framed within a win/loss domain. Thus, we modeled learning from these outcomes with a general extinction rate. It is noteworthy that perseveration is frequently reported in SUD patients (Ersche et al., 2008) and stimulant-exposed animals (Schoenbaum et al., 2004). We would not expect an RL task to be optimized for investigating perseverative responses, unlike a probabilistic reversal learning task (Cools et al., 2002; Jentsch et al., 2002; Schoenbaum et al., 2004; Ersche et al., 2008, 2011a; Kanen et al., 2019). Nevertheless, we included parameters that model perseverative tendencies towards stimuli and locations (i.e., left or right) because accounting for relevant biases might improve model-fit (Wilson and Collins, 2019), as demonstrated in our previous work (Lim et al., 2019). Thus, there were 8 possible parameters in our models: learning rate from reward, non-reward, punishment and non-punishment, general extinction rate, reinforcement sensitivity, as well as perseveration tendencies to stimulus and location, but not all parameters were used in any given model (full details reported in supplementary Table 2). We acknowledge that differences in task designs can change the best-fitting models (Wilson and Collins, 2019), so we fitted several model variants for each study and identified the best-fit model with bridge sampling (Gronau et al., 2017) (supplementary Table 2). To validate the winning model, we simulated data from the winning model to ensure key findings from the actual data were reproduced (supplementary Material).

We estimated the posterior distribution of the best-fit model parameters within a hierarchical Bayesian framework in RStan (Carpenter et al., 2017). In study 1, we modeled a group-level posterior distribution at the top level of the hierarchy for each free parameter. With the inclusion of drug factors in study 2, we constructed group/drug posteriors to model the drug effects on free parameters separately for each group/drug combination. We also constructed a subject-level hierarchy for each parameter to account for any individual variations. Our primary outcome measure was the mean differences between the group/drug posteriors, d, each with its associated 95% highest density intervals (HDI). An HDI interval that excludes zero provides strong evidence for a group difference (non-zero difference, *P*_nz_ > .95).

## Results

Sample characteristics are shown in Table 1. In both studies, the groups were well-matched with respect to age and gender. Verbal intelligence did not differ between the groups in study 2 (t_34_ = −.235, *P* = .816), but SUD patients in sample 1 had lower IQ scores than controls (t_75_ = 8.2, *P* < .001). However, IQ scores in SUD patients were not significantly correlated with learning performance (supplementary Table 3). In both samples, the subjective value of £0.50 did not differ between the groups (study 1: t_83_ = −1.2, *P* = .232; study 2: t_34_ = −1.7, *P* = .098), suggesting that the reinforcement value of monetary rewards was similar in both groups. There were no relationships between learning performance and stimulant-related measures, including the duration or patterns of stimulant use (supplementary Table 3). Consistent with impulsivity being a hallmark of addiction, both patient groups scored significantly higher on the Barratt Impulsiveness Scale-11 compared with controls (study 1: t_83_ = −11.4, *P* < .001; study 2: t_34_ = −7.1, *P* < .001).

### Study 1

#### Conventional Analysis

**Figure 2.**
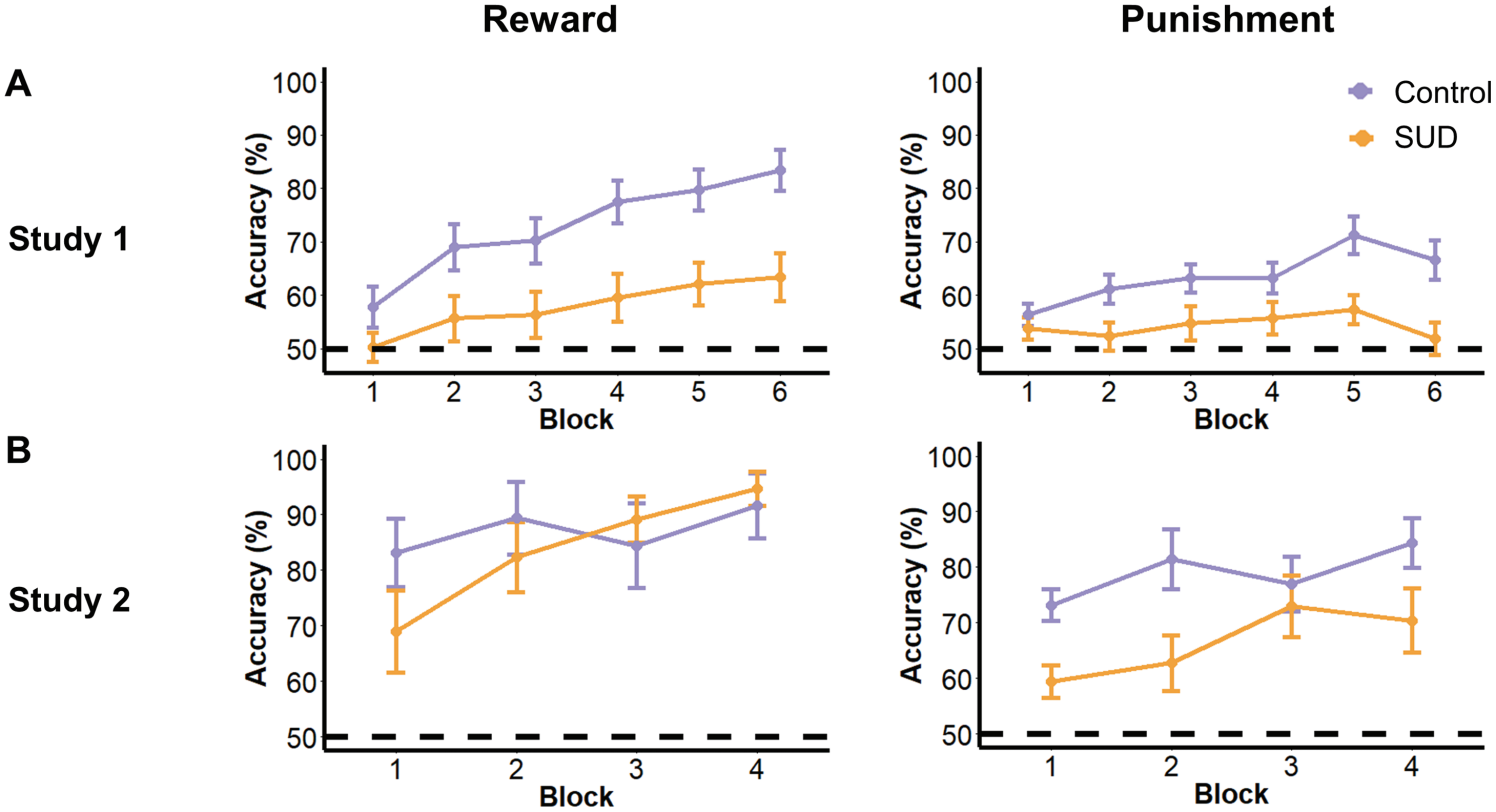
Accuracy scores, defined as the proportion of optimal choices made in 10-trial blocks, for the behavioral task. These scores are plotted separately based on condition (reward and punishment) and group (controls and stimulant use disorder [SUD]). (A) Reinforcement learning performance accuracy in study 1. (B) Reinforcement learning performance accuracy for the placebo condition in study 2. Error bars denote SEM, and the horizontal dotted line indicates accuracy at chance level (50%).

Analyses of accuracy scores showed that there was a main effect of block (F_4.1,341_ = 14.5, *P* < .001) and a block-by-group interaction (F_4.1,341_ = 3.048, *P* = .016), suggesting that although performance improved over time, control participants improved faster than SUD patients. Participants learned faster from reward trials than punishment trials, reflected in a block-by-condition interaction (F_5,415_ = 4.123, *P* = .001) (Figure 2A), but there was no group-by-block-by-condition interaction (F_5,415_ = 0.234, *P* = .948). SUD patients made more errors than controls (F_1,83_ = 18.1, *P* < .001), but no group-by-condition interaction was observed (F_1,83_ = 1.33, *P* = .252).

#### Computational Analysis

**Figure 3.**
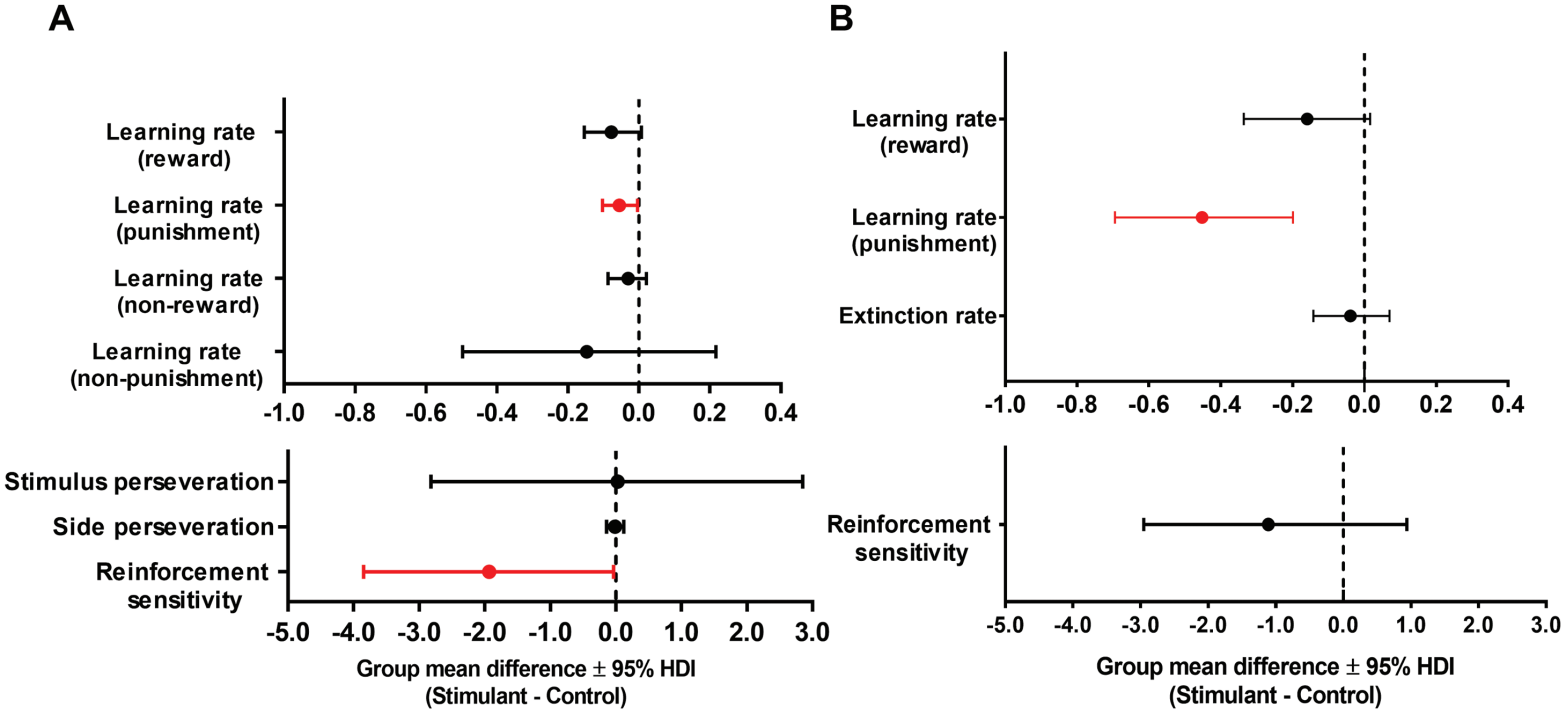
Group mean differences for the reinforcement learning parameters. (A) In study 1, the learning rate from punishment and reinforcement sensitivity were significantly reduced in the stimulant use disorder (SUD) participants, while the other parameters were no different across groups. (B) In the placebo condition of study 2, we found a markedly reduced learning rate from punishment in SUD patients. Error bars denote 95% highest density intervals (HDI); parameters colored in red signify a credible group difference (95% HDI excludes zero).

As shown in Figure 3A, the best-fit learning model contained the following parameters: learning rates from reward, non-reward, punishment and non-punishment, reinforcement sensitivity, and perseveration tendencies toward location and stimulus. SUD patients showed a significantly reduced learning rate from punishment (d = −0.055, 95% HDI = −0.103 to −0.004, *P*_nz_ = .973) and reinforcement sensitivity (d = −1.93, 95% HDI = −3.85 to −0.035, *P*_nz_ = .953). Although the reward learning rate was reduced in SUD patients, the difference was non-significant (d = −0.078, 95% HDI = −0.154 to 0.007, *P*_nz_ = .944) did not differ between groups. The groups did not differ on any other parameters (0 ∈ 95% HDI).

### Study 2

#### Conventional Analysis

On placebo, task performance improved in all participants over time (F_3,102_ = 6.66, *P* < .001), with a significant effect of condition (F_1,34_ = 9.83, *P* = .004) again suggesting that participants learned better from rewarding than punishing feedback (Figure 2B). Control participants learned faster than SUD patients in the first two blocks, as reflected by a significant group-by-block interaction (F_3,102_ = 3.63, *P* = .016). There was neither a group effect (F_1,34_ = 2.52, *P* = .122) nor a group-by-condition interaction  (F_1,34_ = 0.610, *P* = .440). No other effects reached statistical significance (*P*  > .4).

Amisulpride had no significant effect on accuracy (F_1,34_ = .43, *P* = .517), nor were there any group-by-drug interaction effects (F_1,34_ = 0.619, *P* = .437). There was a significant effect of block (F_3,102_ = 18.5, *P* < .001) and condition (F_1,34_ = 15.9, *P* < .001) on accuracy scores, such that all participants showed improved task performance over time and better learning from reward than from punishment. Control participants showed improved accuracy compared with SUD patients (F_1,34_ = 5.41, *P* = .026), but no other effects were significant (all *P* > .1).

Although pramipexole also had a significant effect on accuracy (F_1,31_ = 4.31, *P* = .046), there was a significant drug-by-condition interaction (F_1,31_ = 4.41, *P* = .044). Post-hoc pairwise comparisons revealed a significant reduction of reward relative to punishment trial performance on pramipexole (*P* = .022) but not placebo (*P* = .627). Again, all participants improved performance over time (F_3,93_ = 11.1, *P* < .001), but the effects of condition (F_1,31_ = 2.1, *P* = .157), group (F_1,31_ = 3.41, *P* = .074), and group-by-drug interactions (F_1,34_ = 0.526, *P* = .474) were non-significant. Other effects were also not significant (all *P* > .1).

#### Computational Analysis

The best-fit computational model for study 2 included the following parameters: learning rates from reward and punishment, extinction rate, and reinforcement sensitivity (Figure 3B). On placebo, SUD patients showed markedly reduced rates of learning from punishment (d = −0.452, 95% HDI = −0.695 to −0.199, *P*_nz_ > .999) and marginally reduced learning rate from reward (d = −0.159, 95% HDI = −0.336 to 0.016, *P*_nz_ =929). The groups did not differ in terms of reinforcement sensitivity (d = −1.11, 95% HDI = −2.95 to 0.940, *P*_nz_  = 0.797) or extinction rate (d = −0.039, 95% HDI = −0.142 to 0.070, *P*_nz_  = 0.533).

**Figure 4.**
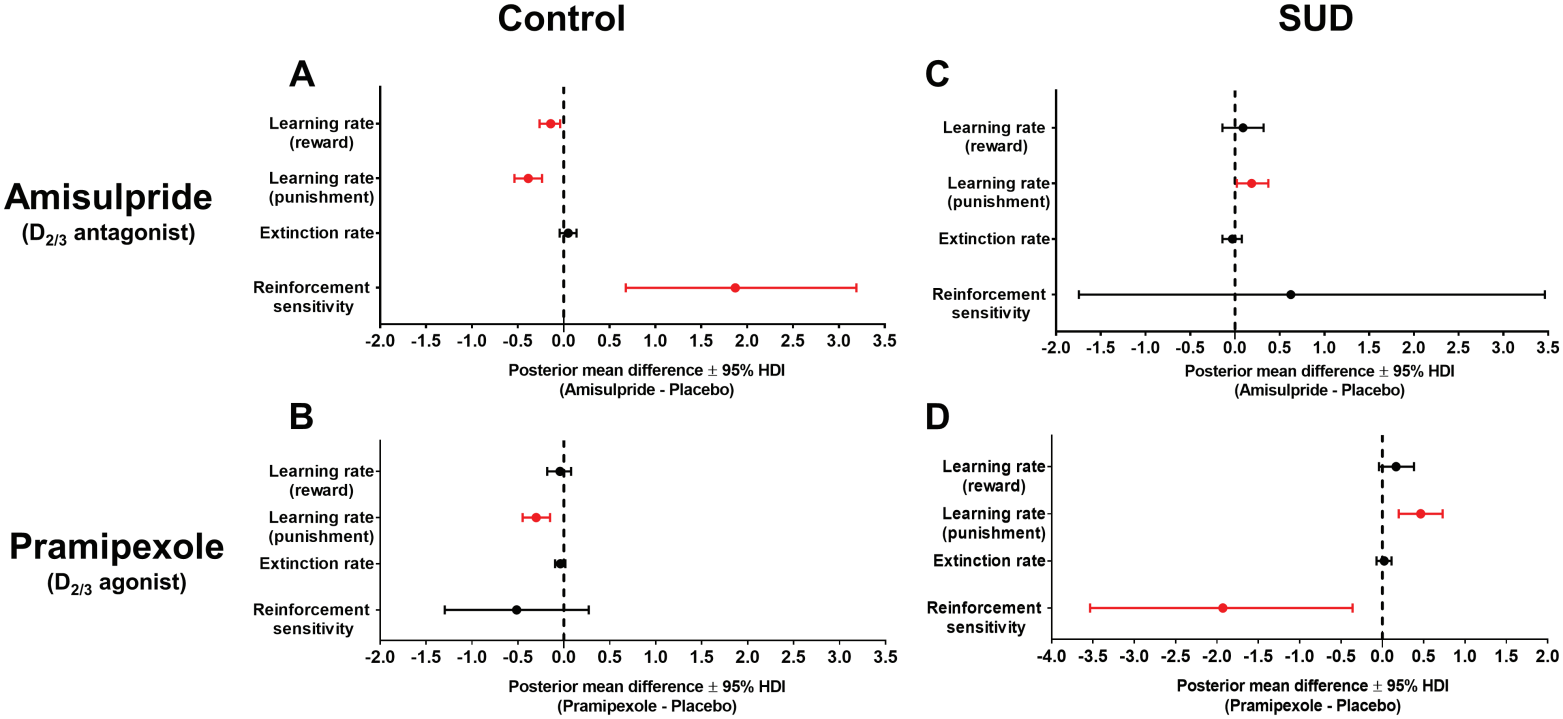
Mean differences of the reinforcement learning parameters for each drug condition. The dopaminergic agents are directly compared with placebo. (A) Amisulpride reduced the learning rates in healthy controls but increased the reinforcement sensitivity parameter. (B) Pramipexole selectively reduced the reward learning rate parameter in control participants, but had no effect on the other parameters. (C) Amisulpride improved the punishment learning rate in stimulant use disorder (SUD) participants. (D) Pramipexole significantly increased punishment learning rate and reduced reinforcement sensitivity parameters in SUD patients. Error bars denote 95% highest density intervals (HDI); parameters colored in red indicate a credible drug effect, as their 95% HDI excludes zero.

Learning parameters were differentially affected by the dopaminergic drugs in both groups. In healthy controls, amisulpride reduced the rates of learning from reward (d = −0.142, 95% HDI = −0.263 to −0.039, *P*_nz_  = 992) and punishment (d = −0.387, 95% HDI = −0.537 to −0.236, *P*_nz_ > .999), and increased reinforcement sensitivity (d = 1.87, 95% HDI = 0.676 to 3.19, *P*_nz_ = .995) (Figure 4A). However, amisulpride improved the rate of learning from punishment in SUD patients (d = 0.186, 95% HDI = 0.020 to 0.373, *P*_nz_ = .975); no other parameters were affected (0 ∈ 95% HDI) (Figure 4C). Similarly, pramipexole reduced punishment learning rates in controls (d = −0.270, 95% HDI = −0.440 to −0.109, *P*_nz_ = .999) (Figure 4B) but improved the punishment learning rate (d = 0.463, 95% HDI = 0.199 to 0.729, *P*_nz_ = .995) in SUD patients. Pramipexole also reduced the reinforcement sensitivity parameter in SUD patients (d = −1.92, 95% HDI = −3.53 to −0.360, *P*_nz_ = .972); other parameters were not affected (0 ∈ 95% HDI) (Figure 4D).

## Discussion

Behavior in SUD patients is thought to be driven by immediate positive outcomes but at the expense of long-term negative consequences (Bechara et al., 2002; Verdejo-Garcia et al., 2018). We investigated RL performance in SUD patients with a task that separately assessed learning from immediate monetary reward and punishment. As hypothesized, computational analyses revealed significant RL impairments in SUD patients, which were driven primarily by a reduced learning rate from punishment. We also found that dopaminergic drugs differentially affected RL parameters in SUD patients and matched controls. While both dopaminergic drugs impaired the learning rates in controls, SUD patients benefitted from them, as both drugs improved their ability to learn from punishment. Here, we provide converging computational and pharmacological evidence of significant learning impairments in SUD patients, which are, at least in part, related to dopamine dysfunction.

### RL Profile in SUD

RL in SUD is characterized by significant impairments in learning from immediate punishment, which may suggest that negative outcomes have little impact on subsequent behavior. This proposal concurs with prior research in animals, demonstrating that psychostimulant self-administration impairs the update of learned values from negative outcomes (Groman et al., 2018, 2020). Moreover, some studies in SUD patients also reported aberrant responses towards immediate negative outcomes, whether those outcomes are electric shocks or symbolic error feedback (Thompson et al., 2012; Hester et al., 2013; Parvaz et al., 2015; Ersche et al., 2016). Negative outcomes such as monetary losses have been suggested to be important in aversive instrumental learning (Jean-Richard-Dit-Bressel et al., 2018). Consequently, the reduced impact of negative feedback during learning may hamper SUD patients’ ability to avoid negative outcomes. From a theoretical perspective, reduced learning from negative consequences may also point towards a weakness in the goal-directed system, which is sensitive to the consequences of one’s actions (Balleine and Dickinson, 1998). In other words, blunted sensitivity towards negative outcomes may weaken the ability to adjust ongoing behavior according to the situational demands and contribute to the development of compulsive behaviors in SUD patients (Smith and Laiks, 2018). The hypothesis of a weakened goal-directed system in SUD is supported by converging lines of evidence in both humans (Lim et al., 2019; Ersche et al., 2020) and animals (Zapata et al., 2010; Corbit et al., 2014).

Although several studies report reduced responses to punishment in SUD patients (Thompson et al., 2012; Hester et al., 2013; Ersche et al., 2016), inconsistent findings have also been observed. For example, a computational analysis by Kanen and colleagues reported increased learning rate for punishment in SUD patients in a serial probabilistic reversal learning task (Kanen et al., 2019). While this task also involves RL, it is important to consider the task context when interpreting these findings. In a probabilistic serial reversal learning task, participants are instructed to expect learned contingencies to change from time to time and thus need to balance between ignoring and responding to punishment, that is, staying with or switching their choices, respectively. An increased learning rate from punishment in this context could thus also reflect an impaired ability to use negative feedback to guide behavior amidst a volatile environment, leading to more errors in SUD patients. Since there were no contingency reversals in our tasks, such divergence in the behavioral profile could be due to intrinsic differences in task design. Indeed, when we fitted the winning model from Kanen et al to the present data, we obtained results consistent with our model—SUD patients still show a reduced learning rate from punishment (supplementary Material).

Compared with learning from punishment, learning from reward was less impaired in SUD patients, indicating that monetary reward remains a salient reinforcer among stimulant drug users. This may suggest that behavior in SUD patients is more amenable to positive than to negative feedback and could explain why treatments based on positive reinforcement such as contingency management (Petry, 2000; Petry et al., 2017) are effective in SUD. Accumulating evidence further suggests that contingency management with monetary incentives is as effective (Festinger et al., 2014), or even more effective (Vandrey et al., 2007; Stoops et al., 2010), in promoting cocaine abstinence and treatment retention than non-monetary incentives (Stitzer et al., 2010). These studies jointly imply that the prospective knowledge of more salient rewards, such as monetary gains, improves contingency learning. Indeed, studies that adopted non-salient feedback (e.g., points or artificial stimuli) in RL tasks reported impairments in learning from reward in SUD patients (Strickland et al., 2016; Lim et al., 2019), possibly reflecting the lack of a motivating reinforcer. This stands in stark contrast to learning from negative consequences, which is significantly impaired regardless of its magnitude (Thompson et al., 2012). However, whether different modes of punishment differentially affect behavior in SUD patients remains an open question.

### Dopaminergic Modulation of RL in Healthy Participants

Although the involvement of dopamine in RL is undisputed, the exact mechanistic role of D_2_ receptors in learning remains controversial, as reflected in the conflicting findings reported in the literature. For example, some studies showed that pharmacological modulation of D_2_ receptors affects only reward but not punishment (Pessiglione et al., 2006; Pizzagalli et al., 2008; Eisenegger et al., 2014), suggesting that D_2_ receptor signaling selectively affects reward learning. However, other evidence from humans (Frank and Hutchison, 2009; Cox et al., 2015) and preclinical studies (Hikida et al., 2010; Kravitz et al., 2012; Alsiö et al., 2019; Verharen et al., 2019) suggests that D_2_ receptor signaling plays a specific role in avoiding negative outcomes (Frank, 2005; Frank and O’Reilly, 2006). While the selective impairment of punishment learning in healthy participants following the D_2/3_ receptor agonist is consistent with the latter view, the observation that the D_2/3_ receptor antagonist affected both reward and punishment does not support the hypothesis that the D_2_ receptor has a valence- specific role in learning. Such non-selective effects of D_2/3_ receptor antagonism have previously been reported (McCabe et al., 2011; Jocham et al., 2014), suggesting that these receptors are generally involved in normal feedback-based learning.

The D_2/3_ receptor antagonist also increased the reinforcement sensitivity parameter in healthy participants, suggesting that amisulpride increased their motivation for higher valued choices. This proposal concurs with other pharmacological studies administering amisulpride, which found that the drug enhanced sensitivity to expected values (Burke et al., 2018) and increased activation during choice selection in the medial-orbitofrontal-cortex (Jocham et al., 2011; Kahnt et al., 2015), a region commonly associated with value representation (O’Doherty, 2004).

When interpreting the drug effects, it is important to consider that dopaminergic D_2/3_ drugs may exert presynaptic actions. At low doses, D_2/3_ agents preferentially bind to pre-synaptic autoreceptors (Schoemaker et al., 1997), which inhibit dopamine transmission (Ford, 2014). Thus, the D_2_ presynaptic autoreceptor blockade by a dopamine antagonist may actually enhance dopamine transmission, whereas stimulation of D_2_ autoreceptors by a dopamine agonist may result in a net reduction of dopaminergic transmission. It is therefore tempting to speculate whether the pramipexole-induced impairments in the learning rate and the amisulpride-induced enhancements in reinforcement sensitivity, as seen in our healthy participants, reflect such pre-synaptic actions.

### Impaired RL Associated With Altered Dopamine System in SUD

The dopaminergic agents had the opposite effect in SUD patients compared with healthy controls, which suggests an altered dopaminergic system in SUD. There is considerable evidence from positron-emission-tomography studies that points toward downregulation of striatal D_2_ receptors and dopaminergic neurotransmission in SUD patients (Volkow et al., 1993, 1997; Martinez et al., 2004, 2007). Repeated stimulant drug exposure has also been proposed to upregulate the inhibitory activity of D_2_ presynaptic autoreceptors, which in turn may suppress dopamine signaling below normal levels (Grace, 1995). However, it is not possible to determine precisely the nature of the dopamine system and the mode of action of the dopamine agents in SUD patients, which depends on dopamine levels at baseline (Cools et al., 2001, 2009). We thus interpreted the effects of dopaminergic agents in light of SUD patients’ possibly reduced dopamine activity and potential pre-synaptic effects of these agents.

If D_2_ receptors are assumed to be important in learning from negative feedback (Frank and O’Reilly, 2006; Nakanishi et al., 2014), the downregulation of D_2_ receptors in SUD would explain their reduced learning from negative outcomes, which is mirrored in healthy individuals with low D_2_ receptor levels (Klein et al., 2007; Jocham et al., 2009). It is therefore conceivable that amisulpride improved punishment learning in SUD by blocking presynaptic D_2_ autoreceptors and thus increasing dopamine signaling. Pramipexole also improved punishment learning in SUD, possibly by enhancing dopamine signaling through post-synaptic mechanisms. It remains, however, unclear why two opposing drugs work in the same direction. It is noteworthy that the reinforcement sensitivity parameter, which measures how much choices are motivated by learned values, was reduced on pramipexole. This may suggest that altering the dopamine balance reduced SUD patients’ tendency to engage in the RL task as the choice values became less motivating. This concurrent reduction in motivation might also explain why SUD patients did not show improvements in overall performance despite an improved punishment learning. The effects of dopaminergic agents seem to confirm altered dopaminergic activity in SUD patients, which have been associated with learning difficulties, but the precise pharmacological actions are likely to depend on task context, drug dosage, and baseline dopamine transmission.

### Strengths, Weaknesses, and Outlook

Our data provide compelling evidence for impaired learning from punishment in two independent samples of SUD patients, one of which had comorbid dependencies while the other had none. Concurrent use of other drugs such as opiate, alcohol, or cannabis is therefore unlikely to have affected the observed performance profiles. Limitations include the uncertainty of the nature of the drug effects, that is, whether they reflect pre-synaptic or post-synaptic effects, which is difficult to determine in human research. Therefore, any inferences on the drug effects should be cautiously interpreted. Further neuroimaging evidence (e.g., positron emission tomography) is warranted to clarify the action of the dopaminergic drugs, as responses to dopaminergic drugs may vary according to baseline dopamine synthesis capacity (Cools et al., 2009) and dopamine receptor density (Cohen et al., 2007; Eisenegger et al., 2014). Although we focused exclusively on dopamine, it is important to acknowledge that other neurotransmitter systems such as serotonin (Seymour et al., 2012) and glutamate (Groman et al., 2020) are also implicated in RL. There is also evidence that amisulpride has an affinity for serotonin receptors (Abbas et al., 2009), which may also modulate sensitivity to aversive events (Daw et al., 2002; Cools et al., 2011). Future studies using a longitudinal design are needed to investigate these factors. Nonetheless, our findings present novel evidence for selective learning impairments in SUD and highlight the utility of computational modeling in deconstructing complex cognitive processes, with promising prospects for psychiatry and psychopharmacology research (Huys et al., 2021).

## Supplementary Material

pyab041__suppl_Supplementary_Material

## Statement of Interest

R.N.C.’s research is funded by the UK Medical Research Council (MC_PC_17213). R.N.C. consults for Campden Instruments Ltd and receives royalties from Cambridge University Press, Cambridge Enterprise, and Routledge. E.T.B. is an NIHR Senior Investigator; he serves on the Scientific Advisory Board of Sosei Heptares and as a consultant for GlaxoSmithKline. T.W.R. discloses consultancy with Cambridge Cognition, Greenfield Bioventures, Heptares, Arcadia, and Takeda; he is a recipient of Wellcome Trust Senior Investigator Award (104631/z/14/z), receives research grants from Shionogi and GlaxoSmithKline, royalties for the Cambridge Neuropsychological Test Automated Battery from Cambridge Cognition, and editorial honoraria from Springer Verlag and Elsevier. K.D.E. was supported by an Alexander von Humboldt Fellowship for senior researchers (GBR 1202805 HFST-E). All authors declare no further conflict of interest.
