# Supplementary material for: Impaired Learning From Negative Feedback in Stimulant Use Disorder: Dopaminergic Modulation
Source: Int J Neuropsychopharmacol. 2021 Jul 1;24(11):867–78. doi: 10.1093/ijnp/pyab041 (PMC8598302; doi:10.1093/ijnp/pyab041)
Supplement: pyab041__suppl_Supplementary_Material [file pyab041__suppl_supplementary_material.pdf]

Supplementary Material:

Impaired learning from negative feedback in stimulant use disorder: Dopaminergic modulation

Tsen Vei Lim, Rudolf N. Cardinal, Edward T. Bullmore, Trevor W. Robbins, Karen D. Ersche

Supplementary methods

Behavioural task: additional description

The probabilistic reinforcement learning tasks were designed to investigate learning from monetary feedback. In both versions, we assessed learning from reward and punishment separately. On each trial, participants were presented with pairs of stimuli and were required to learn by trial and error to select the stimulus that minimizes their financial losses or maximises their winnings. We differentiated between reward and punishment conditions by using distinct stimulus pairs and different feedback type. In the reward condition, the favourable choices received a 'you win 50p' outcome 70% of the time and a 'you win 0p' outcome 30% of the time. By contrast, unfavourable choices were punished (i.e. 'you lose 50p') 70% of the time, and not punished ('you lose 0p') 30% of the time.

As these data were part of two separate larger studies, there were several differences between the task designs:

1 We administered an additional neutral condition to the second sample as a baseline for performance. Like the reward and punishment trials, the neutral trials consisted of a unique stimulus pair, and also maintained a 70% contingency, but choosing either stimuli from this condition had no financial consequences (refer to Figure 1 in manuscript).
2 There were two unique stimulus pairs for each reinforcement condition in study 1 (four in total), but only one unique stimulus pair for each condition in study 2 (three in total).
3 In study 2, no explicit feedback was provided for reward and punishment omission outcomes i.e. only a blank screen.
4 In study 2, we informed participants that their task earnings would be translated to actual monetary bonuses. By contrast, participants did not receive earnings for their performance in study 1, but they were nevertheless instructed to do their best.

Statistical analyses

Computational modelling of behaviour

We modelled the expected values of each choice, trial by trial, using a delta rule (Rescorla and Wagner, 1972):

$$V_{t+1} = V_t + \alpha(R - V_t)$$

V_t denotes the expected value of the chosen stimulus on trial t. The update of this value is determined by the product of α, the learning rate, and the prediction error, the discrepancy between expected value and actual reinforcement received on trial t, R. Mathematically, R is assigned 1 for reward, -1 for punishment, and 0 otherwise. Since there is evidence for different neural systems subserving learning from reward and punishment (Pessiglione and Delgado, 2015), we fractionated α based on the feedback received. In study 1, there are four possible outcomes: reward (i.e. you win 50p), reward omission (i.e. you win 0p), punishment (i.e. you lose 50p) and punishment omission (i.e. you lose 0p), hence the learning from each outcome was modelled with a different α as follows:

$V_{t+1} = V_t + \alpha_{rew}(R - V_t)$ if feedback = “You win 50p”;

$V_{t+1} = V_t + \alpha_{non-rew}(R - V_t)$ if feedback = “You win 0p”;

$V_{t+1} = V_t + \alpha_{pun}(R - V_t)$ if feedback = “You lose 50p”;

$V_{t+1} = V_t + \alpha_{non-pun}(R - V_t)$ if feedback = “You lose 0p”;

where α_{rew}, $\alpha_{non-rew}$, α_{pun}, and $\alpha_{non-pun}$, refers to the learning rates from reward, non-reward, punishment, and non-punishment respectively.

In study 2, reward and punishment omission outcomes were not explicitly framed within a win/loss domain; instead participants were not provided with explicit feedback (Figure 1 from manuscript). Therefore, it would not be apparent from this feedback whether participants experienced a loss/gain

from their selection. Thus, we modelled learning from these outcomes with a general extinction rate, α_{ext}:

$$V_{t+1} = V_t + \alpha_{rew}(R - V_t) \text{ if feedback = "You win 50p";}$$

$$V_{t+1} = V_t + \alpha_{pun}(R - V_t) \text{ if feedback = "You lose 50p";}$$

$$V_{t+1} = V_t + \alpha_{ext}(R - V_t) \text{ if no feedback given;}$$

Increases in α would indicate an increased rate of stimulus value update from the corresponding feedback.

We used the expected values to model actual choice behaviour, via a softmax rule:

$$p(i,t) = \frac{exp(\beta V_t^i)}{\sum_{k=1}^{n} exp(\beta V_t^k)}$$

This equation gives the model's probability of choosing choice i amongst n choices on a trial t. The extent to which expected values are used to drive choices is governed by the reinforcement sensitivity parameter, β. Cocaine use disorder has been associated with the tendency to perseverate responses irrespective of reinforcement (Ersche et al., 2008, 2011), and this may affect learning. Hence, in models where we accounted for perseveration, we modified the softmax equation to include the term C_{stim} and C_{loc} to indicate perseveration towards the previously chosen stimulus and towards the previously chosen location (e.g. left, right) respectively, regardless of choice value:

$$p(i,t) = \frac{exp\left(\beta V_t^i + \tau_{stim} C_{stim}{}_t^i + \tau_{loc} C_{loc}{}_t^i\right)}{\sum_{k=1}^{n} exp\left(\beta V_t^k + \tau_{stim} C_{stim}{}_t^k + \tau_{loc} C_{loc}{}_t^k\right)}$$

C_{stim} and C_{loc} are assigned 1 if participants repeated their choices for the same stimulus (e.g. chose stimulus A on previous trial, and choose stimulus A again on current trial) and location (e.g. responded 'left' last trial, and respond with 'left' again this trial) respectively, and 0 if choices were not repeated. The tendency for perseveration to influence choice is governed by perseveration parameters for stimulus, τ_{stim}, and for location, τ_{stim}. There were in total eight possible free parameters in our learning models (not

all used in any given model): learning rate from reward, non-reward, punishment and non-punishment, general extinction rate, reinforcement sensitivity, and well as perseveration tendencies to stimulus and side. We used bridge sampling procedures to identify the best-fitting model from several variants of our learning models (see section below and Table S2).

Parameter estimation

We estimated the posterior distribution of free parameters by analysing the best-fit model within a hierarchical Bayesian framework. For each parameter, we modelled a group-level distribution at the top of the hierarchy. For study 2, we used each group/drug combination. The posterior distributions of group-level (or group/drug) parameters were our main measures of interest. Prior distributions were assigned to all parameters (see Table S1). We also modelled inter-subject variability and accounted for the within-subject aspects of the design: for each parameter, subject-specific deviations from the group mean were drawn from a normal distribution with mean 0 and a parameter-specific standard deviation (itself estimated). Subject-specific parameters were then used in the reinforcement learning model to predict choice (what does the model predict is the probability of the subject choosing stimulus A?), and the model was fitted by comparing these to actual choices made (did the subject choose stimulus A?). We implemented the procedure in RStan (version 2.17.2), which uses a Markov chain Monte Carlo approach. We simulated eight parallel chains, each with 2000 iterations (including warmup), and directly sampled posterior distributions of the group/drug mean differences, d, as the primary outcome measure. Measures of dispersion for the group differences are indicated by the 95% highest density intervals (HDI); a 95% HDI that excludes zero provides strong evidence for a group difference (non-zero-difference, $p_{nz}>0.95$). No multiple comparison corrections were applied, because Bayesian hierarchical analyses tend to produce more conservative comparisons by shifting point estimates towards each other (‘partial pooling’), making intervals more likely to include zero (Gelman et al., 2012).

Model selection

We used bridge sampling, implemented via the "*bridgesampling*" package in R, to determine the best-fit model. Given that the correct model is one of the models being considered, the posterior probability of a model, *P*(model | data), can be measured directly by taking (1) the prior probability of the model itself, *P*(model), and (2) the marginal likelihood of, or evidence for, the model, *P*(data | model), which can be estimated via bridge sampling. The marginal likelihood integrates, over all possible parameter values, the product of (2a) the likelihood of the data given the fitted model (how well the model fits the data), *P*(data | parameters, model), and (2b) the probability of the parameters given the model, *P*(parameters | model), thus incorporating Occam's razor by penalizing over-complex models. We assumed all models were equiprobable *a priori*. We also report the Bayes factor, defined as the ratios of marginal likelihood of a pair of models, as a secondary indicator for model evidence. Model comparison results are reported in Table S2. Generally, a Bayes factor of more than one constitutes sufficient evidence that one model is better than the next (Kass and Raftery, 1995).

Winning model validation

It is important that the winning model is able to recover key aspects of the behavioural findings (Wilson and Collins, 2019). To that end we performed a posterior predictive check by simulating data for 50 'virtual' subjects per group for each study, using the posterior mean values for each group-level parameter. We did not incorporate subject variance, as we were interested in group effects instead of individual variability. The simulated data were analysed using analysis of variance (ANOVA) in the same way as reported in the main manuscript text, with block and condition (reward versus punishment) as within-subject factors, and group (SUD versus controls) as a between-subjects factor.

As an extra validation step, we also fitted our data to the winning model of Kanen et al., who recently analysed behavioural performance of stimulant use disorder (SUD) and obsessive-compulsive disorder (OCD) patients on a serial probabilistic reversal learning task (Kanen et al., 2019). The main purpose of

this analysis is to determine whether the divergence in our results and Kanen et al.'s, specifically in the punishment learning rate, is due to intrinsic differences in our behavioural tasks or modelling procedures. The winning model in Kanen et al. had five parameters: reward learning rate, punishment learning rate, reinforcement sensitivity, stimulus stickiness and location stickiness (Kanen et al., 2019). The main difference between our winning models and that of Kanen et al.'s is our inclusion of extinction rate parameters into the models i.e. we included parameters that modelled separately the effects of reward omission and punishment avoidance events. This method of modelling was not possible in Kanen et al's study as their task only contained two types of feedback (reward and non-reward). Thus, to mimic Kanen et al's winning model as closely as possible, we removed the extinction rate parameters and modelled all reward trials (reward delivery + omission) with the reward learning rate, and all punishment trials (punishment + avoidance) with the punishment learning rate. The marginal likelihood for this model was also estimated with bridge sampling to compare it against our winning model. If this analysis produce results consistent with our winning model, it would suggest that any intrinsic differences between our findings and that of Kanen et al's are likely attributed to difference between behavioural tasks (e.g. the inclusion of reversals); if not, then the divergence with our results might be a product of different modelling procedures.

Supplementary results

Model selection and winning model validation

The winning model in study 1 included the following parameters: learning rates from reward, punishment, non-reward, and non-punishment; perseveration towards location and stimulus; and reinforcement sensitivity. Based on the criteria of Kass and Raftery (1995), there was overwhelming evidence that the winning model is superior to the next best model (Table S2). The winning model for study 2 consisted of four parameters: learning rates from reward and punishment, extinction rate, and reinforcement sensitivity. Again the Bayes factor suggests that the winning model was far superior to the next-ranked model.

We were able to validate the winning models for each study. For study 1, analyses on simulated data from the winning model were able to recover the main effects of group ($F_{1,98}$=38.5,p<.001), block ($F_{5,490}$=60.3, p<0.001), condition ($F_{1,98}$=91.1, p<0.001) as well as the lack of group-by-condition interaction ($F_{1,98}$=1.93, p=0.168) as reported in the manuscript. For study 2, analyses on accuracy scores of simulated data from placebo recovered the main effect of condition ($F_{1,98}$=64.4, p<0.001), block ($F_{3,294}$=22.7, p<.001), and group-by-block interaction ($F_{3,294}$=4.22, p=.006), and the lack of group effect ($F_{1,98}$=0.666, p=.416) and group-by-condition interaction ($F_{1,98}$=1.05, p=.308). Simulation of amisulpride data was also able to reproduce all the main effects: we were able to recover the main effects of group ($F_{1,98}$=9.06, p=.003), condition ($F_{1,98}$=61.1, p<.001), block ($F_{3,294}$=93.9, p<.001) as well as the lack of drug effect ($F_{1,98}$=1.10, p=.297). The simulated data additionally yielded a group-by-drug interaction ($F_{1,98}$=15.3, p<.001) such that amisulpride significantly reduced accuracy in SUD patients (t_{98}=3.81, p=.001), but not controls (t_{98}=2.2, p=.123); this difference could be due to the removal of participant variability in the simulated data, which might have increased the effect size. Simulations of pramipexole data recaptured the main effect of block ($F_{3,294}$=88, p<.001) and drug-by-condition interaction ($F_{1,98}$=5.82, p=.018). The simulated pramipexole data also found a main effect of condition

($F_{1,98}$=84, p<.001), which again may be due to reduced variance in simulated data; all other effects were not statistically significant (p > 0.1).

We analysed our data with the winning model in Kanen et al. as an extra confirmatory step. It is worth noting that bridge sampling procedures indicate that this model was ranked the 6th best model in both studies (Table S2), which means that results from this model do not best describe our data. Nevertheless, analyses with Kanen et al.'s winning model replicated our main finding for the reduced punishment learning rate in both study 1 (d = -0.064, 95% HDI = -0.118 to -0.007, p_{nz} = 0.972) and the placebo condition in study 2 (d = -0.182, 95% HDI = -0.305 to -0.055, p_{nz} = 0.995). The ability to replicate the reduced punishment learning rate with Kanen et al's winning model confirms that the divergent findings on punishment-driven learning are due to intrinsic differences between our RL task and one with contingency reversals.

Additional correlations between demographics and task performance

Since there was a group difference in verbal IQ in study 1, as measured with the National Adult Reading Test (Nelson, 1982), we further assessed the relationship between verbal IQ and task performance in the SUD group. We also investigated the relationship between task performance measures and stimulant use duration, as well as severity of compulsive drug use as measured by the Obsessive-Compulsive Drug Use Scale (OCDUS). Results are shown in Table S3. We did not find any significant correlations between these demographic measures and task performance in both studies, whether measured by accuracy scores or inferred computational learning parameters.

Supplementary Tables and Figures

Table S1: Prior distributions for all possible parameters.
*all standard deviation priors are constrained to be positive.

Parameter	Range (lower bound, upper bound)	Group mean priors	Inter-subject standard deviation priors*
Reward learning rate	0, 1	Beta(1.1, 1.1)	Normal(0, 0.05)
Punishment learning rate	0, 1	Beta(1.1, 1.1)	Normal(0, 0.05)
Non-reward learning rate	0, 1	Beta(1.1, 1.1)	Normal(0, 0.05)
Non-punishment learning rate	0, 1	Beta(1.1, 1.1)	Normal(0, 0.05)
General learning rate	0, 1	Beta(1.1, 1.1)	Normal(0, 0.05)
General extinction rate	0, 1	Beta(1.1, 1.1)	Normal(0, 0.05)
Reinforcement sensitivity	0, ∞	Gamma(α = 4.82, β = 0.88)	Normal(0, 1)
Perseveration towards location (side)	-∞, +∞	Normal(0, 1)	Normal(0, 0.05)
Perseveration towards stimulus	-∞, +∞	Normal(0, 1)	Normal(0, 0.05)

Table S2: Variants of learning models and model comparison results. Unless otherwise stated, log refers to the natural logarithm. [Note: α_{rew}: learning rate from reward; $\alpha_{non\text{-}rew}$: learning rate from non-reward; α_{pun}: learning rate from punishment; $\alpha_{non\text{-}pun}$: learning rate from non-reward; α: general learning rate; α_{ext}: extinction rate; β: reinforcement sensitivity; τ_{loc}: perseveration by location ("side"); τ_{stim}: perseveration by stimulus]

Model parameters	Ranking	Log marginal likelihood	Log posterior p(model)	Posterior p(model)	Log_{10} Bayes Factor (relative to next-ranked model)
Study 1					
**$\alpha_{rew}, \alpha_{non\text{-}rew}, \alpha_{pun}, \alpha_{non\text{-}pun}, \beta, \tau_{loc}, \tau_{stim}$**	**1**	**-5586.47**	**-0.009**	**0.991**	**2.058**
$\alpha_{rew}, \alpha_{non\text{-}rew}, \alpha_{pun}, \alpha_{non\text{-}pun}, \beta, \tau_{loc}$	2	-5591.21	-4.747	0.009	7.698
$\alpha_{rew}, \alpha_{non\text{-}rew}, \alpha_{pun}, \alpha_{non\text{-}pun}, \beta, \tau_{stim}$	3	-5608.93	-22.5	1.74×10^{-10}	0.069
$\alpha_{rew}, \alpha_{non\text{-}rew}, \alpha_{pun}, \alpha_{non\text{-}pun}, \beta$	4	-5609.09	-22.6	1.49×10^{-10}	8.385
$\alpha_{rew}, \alpha_{pun}, \beta, \tau_{loc}$	5	-5628.40	-41.9	6.12×10^{-19}	0.870
$\alpha_{rew}, \alpha_{pun}, \beta, \tau_{loc}, \tau_{stim}$	6	-5630.40	-43.9	8.27×10^{-20}	6.831
$\alpha_{rew}, \alpha_{pun}, \beta, \tau_{stim}$	7	-5646.13	-59.7	1.22×10^{-26}	0.013
$\alpha_{rew}, \alpha_{pun}, \beta$	8	-5646.16	-59.7	1.19×10^{-26}	9.389
$\alpha_{rew}, \alpha_{pun}, \alpha_{ext}, \beta, \tau_{loc}$	9	-5667.78	-81.3	4.84×10^{-36}	1.323
$\alpha_{rew}, \alpha_{pun}, \alpha_{ext}, \beta, \tau_{loc}, \tau_{stim}$	10	-5670.82	-84.3	2.30×10^{-37}	9.389
$\alpha_{rew}, \alpha_{pun}, \alpha_{ext}, \beta, \tau_{stim}$	11	-5692.44	-105.9	9.39×10^{-47}	0.005
$\alpha_{rew}, \alpha_{pun}, \alpha_{ext}, \beta$	12	-5692.45	-106.0	9.29×10^{-47}	87.24
$\alpha, \beta, \tau_{loc}, \tau_{stim}$	13	-5893.33	-306.9	5.37×10^{-134}	0.851
$\alpha, \beta, \tau_{loc}$	14	-5895.29	-308.8	7.57×10^{-135}	6.777
α, β	15	-5910.89	-324.4	1.26×10^{-141}	0.083
$\alpha, \beta, \tau_{stim}$	16	-5911.08	-324.6	1.04×10^{-141}	131.5
$\alpha_{rew}, \alpha_{non\text{-}rew}, \alpha_{pun}, \alpha_{non\text{-}pun}, \tau_{loc}, \tau_{stim}$	17	-6213.89	-627.4	3.24×10^{-273}	0.909
$\alpha_{rew}, \alpha_{non\text{-}rew}, \alpha_{pun}, \alpha_{non\text{-}pun}$	18	-6215.98	-629.5	4.00×10^{-274}	33.40
$\alpha_{rew}, \alpha_{pun}, \tau_{loc}, \tau_{stim}$	19	-6292.88	-706.4	1.61×10^{-307}	2.452
$\alpha_{rew}, \alpha_{pun}, \alpha_{ext}, \tau_{loc}, \tau_{stim}$	20	-6298.52	-712.1	5.68×10^{-310}	5.742
$\alpha_{rew}, \alpha_{pun}, \alpha_{ext}$	21	-6311.75	-725.3	1.03×10^{-315}	40.53
$\alpha, \tau_{loc}, \tau_{stim}$	22	-6405.06	-818.6	$< 5 \times 10^{-324}$	288.8
None (random choice model)	23	-7070.10	-	-	-
Study 2					
**$\alpha_{rew}, \alpha_{pun}, \alpha_{ext}, \beta$**	**1**	**-5887.29**	**-0.002**	**0.998**	**2.768**
$\alpha_{rew}, \alpha_{pun}, \alpha_{ext}, \beta, \tau_{stim}$	2	-5893.67	-6.374	0.002	3.201
$\alpha_{rew}, \alpha_{pun}, \alpha_{ext}, \beta, \tau_{stim}, \tau_{loc}$	3	-5901.04	-13.75	1.07×10^{-6}	1.032
$\alpha_{rew}, \alpha_{pun}, \alpha_{ext}, \beta, \tau_{loc}$	4	-5903.42	-16.13	9.91×10^{-8}	33.96
$\alpha_{rew}, \alpha_{pun}, \beta$	5	-5981.62	-94.33	1.08×10^{-41}	4.093
$\alpha_{rew}, \alpha_{pun}, \beta, \tau_{stim}, \tau_{loc}$	6	-5991.04	-103.75	8.73×10^{-46}	1.656
$\alpha_{rew}, \alpha_{pun}, \beta, \tau_{loc}$	7	-5994.86	-107.6	1.93×10^{-47}	5.029
α, β	8	-6006.44	-119.1	1.80×10^{-52}	6.635
$\alpha, \beta, \tau_{loc}$	9	-6021.71	-134.4	4.18×10^{-59}	450.6
$\alpha_{rew}, \alpha_{pun}, \alpha_{ext}, \tau_{stim}$	10	-7059.22	-1171.9	$< 5 \times 10^{-324}$	0.012
$\alpha_{rew}, \alpha_{pun}, \alpha_{ext}$	11	-7059.25	-1171.0	$< 5 \times 10^{-324}$	6.414
$\alpha_{rew}, \alpha_{pun}, \alpha_{ext}, \tau_{stim}, \tau_{loc}$	12	-7074.02	-1186.7	$< 5 \times 10^{-324}$	4.869
$\alpha_{rew}, \alpha_{pun}, \alpha_{ext}, \tau_{loc}$	13	-7085.23	-1197.9	$< 5 \times 10^{-324}$	608.7
None (random choice model)	14	-8486.89	-	-	-

Table S3: Correlations between demographics and task performance in stimulant use disorder patients. None of the pairwise correlations were statistically significant. [NART: National Adult Reading Test; OCDUS: Obsessive-Compulsive Drug Use Scale]

Task performance measure	Verbal IQ (NART score)		Duration of stimulant use (years)		Compulsive drug use severity (OCDUS score)	
Study 1						
Conventional measures	r	p	r	p	r	p
Accuracy score (reward)	-.130	.939	-.166	.281	-.067	.664
Accuracy score (punishment)	.076	.647	-.157	.310	-.211	.169
Computational parameters	r	p	r	p	r	p
Learning rate from reward	.106	.521	-.209	.173	-.141	.361
Learning rate from punishment	-.039	.812	-.194	.206	-.284	.061
Learning rate from non-reward	-.091	.580	-.005	.975	-.111	.474
Learning rate from non-punishment	.122	.458	-.169	.272	-.185	.231
Reinforcement sensitivity	.146	.377	-.140	.366	-.010	.950
Perseveration by location	-.257	.114	.263	.084	.057	.714
Perseveration by stimulus	-.204	.214	-.002	.989	-.031	.839
Study 2 (placebo)						
Conventional measures	r	p	r	p	r	p
Accuracy score (reward)	.003	.991	-.086	.734	-.253	.311
Accuracy score (punishment)	-.179	.476	-.240	.337	.298	.230
Computational parameters	r	p	r	p	r	p
Learning rate from reward	.066	.795	-.457	.057	-.255	.308
Learning rate from punishment	.262	.294	.013	.959	.323	.192
General extinction rate	.196	.435	.054	.832	.124	.625
Reinforcement sensitivity	.192	.446	.053	.834	-.128	.614

Figure S1: Group posterior distributions for each parameter of the winning model. (a) Group posteriors for study 1. (b) Group posteriors for the placebo condition in study 2. [Note: SUD: stimulant use disorder; α_{rew}: learning rate from reward; $\alpha_{non\text{-}rew}$: learning rate from non-reward; α_{pun}: learning rate from punishment; $\alpha_{non\text{-}pun}$: learning rate from non-reward; α_{ext}: general extinction rate; β: reinforcement sensitivity; τ_{loc}: perseveration by location ("side"); τ_{stim}: perseveration by stimulus]

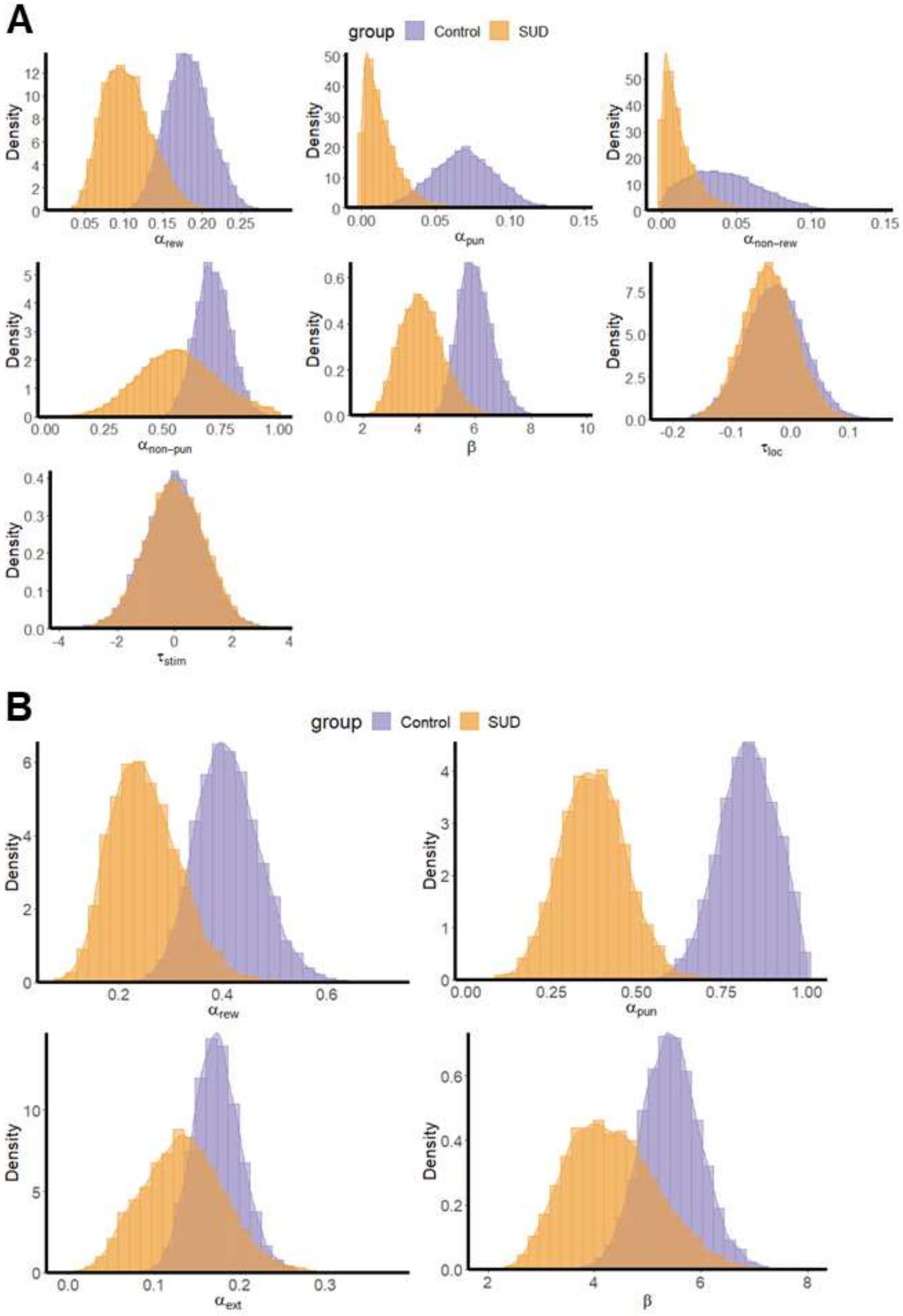

Supplementary References

Ersche KD, Roiser JP, Abbott S, Craig KJ, Müller U, Suckling J, Ooi C, Shabbir SS, Clark L, Sahakian BJ, Fineberg NA, Merlo-Pich EV, Robbins TW, Bullmore ET (2011) Response Perseveration in Stimulant Dependence Is Associated with Striatal Dysfunction and Can Be Ameliorated by a D2/3 Receptor Agonist. Biol Psychiatry 70:754–762.

Ersche KD, Roiser JP, Robbins TW, Sahakian BJ (2008) Chronic cocaine but not chronic amphetamine use is associated with perseverative responding in humans. Psychopharmacology (Berl) 197:421–431.

Gelman A, Hill J, Yajima M (2012) Why We (Usually) Don’t Have to Worry About Multiple Comparisons. J Res Educ Eff 5:189–211.

Kanen JW, Ersche KD, Fineberg NA, Robbins TW, Cardinal RN (2019) Computational modelling reveals contrasting effects on reinforcement learning and cognitive flexibility in stimulant use disorder and obsessive-compulsive disorder: remediating effects of dopaminergic D2/3 receptor agents. Psychopharmacology (Berl) 236:2337–2358.

Kass RE, Raftery AE (1995) Bayes Factors. J Am Stat Assoc 90:773–795.

Nelson HE (1982) National adult reading test (NART). Windsor: Nfer-Nelson Windsor.

Pessiglione M, Delgado MR (2015) The good, the bad and the brain: neural correlates of appetitive and aversive values underlying decision making. Curr Opin Behav Sci 5:78–84.

Rescorla RA, Wagner AR (1972) A Theory of Pavlovian Conditioning: Variations in the Effectiveness of Reinforcement and Nonreinforcement. In: Classical Conditioning II: Current Research and Theory (Black AH, Prokasy WF, eds), pp 64–99. New York: Appleton Century Crofts.

Wilson RC, Collins AG (2019) Ten simple rules for the computational modeling of behavioral data Behrens TE, ed. eLife 8:e49547.
